# Genome Sequence of Babesia bovis and Comparative Analysis of Apicomplexan Hemoprotozoa

**DOI:** 10.1371/journal.ppat.0030148

**Published:** 2007-10-19

**Authors:** Kelly A Brayton, Audrey O. T Lau, David R Herndon, Linda Hannick, Lowell S Kappmeyer, Shawn J Berens, Shelby L Bidwell, Wendy C Brown, Jonathan Crabtree, Doug Fadrosh, Tamara Feldblum, Heather A Forberger, Brian J Haas, Jeanne M Howell, Hoda Khouri, Hean Koo, David J Mann, Junzo Norimine, Ian T Paulsen, Diana Radune, Qinghu Ren, Roger K Smith, Carlos E Suarez, Owen White, Jennifer R Wortman, Donald P Knowles, Terry F McElwain, Vishvanath M Nene

**Affiliations:** 1 Program in Genomics, Department of Veterinary Microbiology and Pathology, Washington State University, Pullman, Washington, United States of America; 2 Animal Disease Research Unit, United States Department of Agriculture, Agricultural Research Service, Pullman, Washington, United States of America; 3 The Institute for Genomic Research, Rockville, Maryland, United States of America; 4 Division of Cell and Molecular Biology, Faculty of Life Sciences, Imperial College, London, United Kingdom; New York University School of Medicine, United States of America

## Abstract

Babesia bovis is an apicomplexan tick-transmitted pathogen of cattle imposing a global risk and severe constraints to livestock health and economic development. The complete genome sequence was undertaken to facilitate vaccine antigen discovery, and to allow for comparative analysis with the related apicomplexan hemoprotozoa Theileria parva and Plasmodium falciparum. At 8.2 Mbp, the B. bovis genome is similar in size to that of Theileria spp. Structural features of the B. bovis and T. parva genomes are remarkably similar, and extensive synteny is present despite several chromosomal rearrangements. In contrast, B. bovis and P. falciparum, which have similar clinical and pathological features, have major differences in genome size, chromosome number, and gene complement. Chromosomal synteny with P. falciparum is limited to microregions. The B. bovis genome sequence has allowed wide scale analyses of the polymorphic variant erythrocyte surface antigen protein (*ves1* gene) family that, similar to the *P. falciparum var* genes, is postulated to play a role in cytoadhesion, sequestration, and immune evasion. The ∼150 *ves1* genes are found in clusters that are distributed throughout each chromosome, with an increased concentration adjacent to a physical gap on chromosome 1 that contains multiple *ves1*-like sequences. *ves1* clusters are frequently linked to a novel family of variant genes termed *smorf*s that may themselves contribute to immune evasion, may play a role in variant erythrocyte surface antigen protein biology, or both. Initial expression analysis of *ves1* and *smorf* genes indicates coincident transcription of multiple variants. B. bovis displays a limited metabolic potential, with numerous missing pathways, including two pathways previously described for the P. falciparum apicoplast. This reduced metabolic potential is reflected in the B. bovis apicoplast, which appears to have fewer nuclear genes targeted to it than other apicoplast containing organisms. Finally, comparative analyses have identified several novel vaccine candidates including a positional homolog of p67 and SPAG-1, *Theileria* sporozoite antigens targeted for vaccine development. The genome sequence provides a greater understanding of B. bovis metabolism and potential avenues for drug therapies and vaccine development.

## Author Summary

Vector-transmitted blood parasites cause some of the most widely distributed, serious, and poorly controlled diseases globally, including the most severe form of human malaria caused by Plasmodium falciparum. In livestock, tick-transmitted blood parasites include the protozoa Theileria parva, the cause of East Coast fever and Babesia bovis, the cause of tick fever, to which well over half of the world's cattle population are at risk. There is a critical need to better understand the mechanisms by which these parasites are transmitted, persist, and cause disease in order to optimize methods for control, including development of vaccines. This manuscript presents the genome sequence of B. bovis, and provides a whole genome comparative analysis with P. falciparum and T. parva. Genome-wide characterization of the B. bovis antigenically variable *ves1* family reveals interesting differences in organization and expression from the related *P. falciparum var* genes. The second largest gene family (*smorf*) in B. bovis was newly discovered and may itself be involved in persistence, highlighting the utility of this approach in gene discovery. Organization and structure of the B. bovis genome is most similar to that of *Theileria*, and despite common features in clinical outcome is limited to microregional similarity with P. falciparum. Comparative gene analysis identifies several previously unknown proteins as homologs of vaccine candidates in one or more of these parasites, and candidate genes whose expression might account for unique properties such as the ability of *Theileria* to reversibly transform leukocytes.

## Introduction

Babesiosis is a tick-borne, hemoprotozoan disease enzootic in ruminants in most sub-temperate and tropical areas of the world (reviewed in [1]). It is recognized as an emerging zoonotic disease of humans, particularly in immunocompromised individuals [2], and is of historical significance as the first protozoan agent recognized to be arthropod transmitted [3]. With no widely available vaccine and a nearly global distribution, babesiosis is one of the most important arthropod-transmitted diseases of cattle, with over half of the world's cattle population at risk [4]. Live attenuated vaccines are used for the control of babesiosis in many parts of the world, but rely on region-specific attenuated strains for which vaccine breakthrough is not uncommon (reviewed in [5]). Due to the blood-based production of these attenuated vaccines and the possibility of reversion to virulence with tick passage, they are not licensed in the US. The consequences of a disease outbreak in a naïve cattle population with no available vaccine would be catastrophic.

*Babesia*, the causative agent of babesiosis, is in the order Piroplasmida within the phylum Apicomplexa [6]. Similar to other members of this phylum, such as the phylogenetically closely positioned *Theileria* and its distant cousin, *Plasmodium*, *Babesia* undergoes a complex life cycle that involves both vector and mammalian hosts. In contrast to *Plasmodium*, for which *Anopheles* mosquitoes vector transmission, *Theileria* and *Babesia* are transmitted via tick vectors. For all three hemoprotozoans, sporozoites are injected into the blood stream of the mammalian host and it is at this stage where the life cycle of *Babesia* differs from that of *Theileria* and *Plasmodium*. For *Theileria*, infection leads first to lymphocytic stages followed after schizogony by intraerythrocytic development [7]. In plasmodial infection, the sporozoite first infects hepatocytes in which the stage infecting the erythrocytes is produced. In contrast, babesial infection with sporozoites leads directly to infection of erythrocytes. Once inside an erythrocyte, both *Theileria* and *Babesia* are found in the cytoplasm while *Plasmodium* resides in a parasitophorous vacuole. In spite of the differences in the mammalian cell types that the parasites invade, the hallmarks of a B. bovis–induced clinical syndrome in cattle, including severe anemia, capillary sequestration of infected erythrocytes, abortion, and a neurologic syndrome, are remarkably similar to human malaria caused by Plasmodium falciparum [8,9]. Whether the mechanisms leading to these clinical features are unique or are shared between these two related hemoprotozoans is unknown.

Complete apicomplexan genome sequences for T. parva, T. annulata, and P. falciparum have been reported [7,10,11]. Comparisons of these genomes revealed that only approximately 30%–38% of the predicted proteins could be assigned a function, suggesting that the majority of the proteins for these organisms are novel [10,11]. Data from the genome sequences demonstrate many differences between *Plasmodium* and *Theileria*, such as the number of rRNA units and their developmental regulation, the lack of key enzymes in certain metabolic pathways, lengths of intergenic regions, gene density, and intron distribution. The genome sequence of the virulent, tick-transmissible Texas T2Bo isolate of B. bovis, reported here, will allow for an even more comprehensive, genome-wide comparison of this triad of important vector-borne apicomplexan hemoprotozoa, and can be used to identify genes that play common and species-specific roles in apicomplexan biology. Furthermore, insight from such comparisons may improve our ability to design potential prophylactic and therapeutic drug targets.

## Results/Discussion

### Genome Structure and Sequence

Assembly of whole genome shotgun sequence data of the Texas T2Bo isolate of B. bovis indicates that the parasite contains four chromosomes, confirming previous results from pulse field gel electrophoresis [12,13]. Chromosome 1, the smallest of the four chromosomes, contains a large physical gap flanked by two large contigs (821,816 bp and 285,379 bp in length). The gap is estimated to be 150 kbp by pulse field gel electrophoresis (unpublished data) and contains five contigs that vary in size from 12 kbp to 28 kbp, with the order of the contigs in the gap unknown. Chromosomes 2 and 3 were fully sequenced and are 1,729,419 and 2,593,321 bp in length, respectively. Chromosome 4 contains an assembly gap that has not been unambiguously resolved; a 1,149 bp contig separates two contigs of 827,912 bp and 1,794,700 bp. Thus, the nuclear genome of B. bovis consists of four chromosomes of 2.62, 2.59, 1.73, and ∼1.25 Mbp in length. At 8.2 Mbp in size, the genome of B. bovis is similar in size to that of T. parva (8.3 Mbp) [10] and T. annulata (8.35 Mbp) [7], the smallest apicomplexan genomes sequenced to date (Table 1).

**Table 1:**
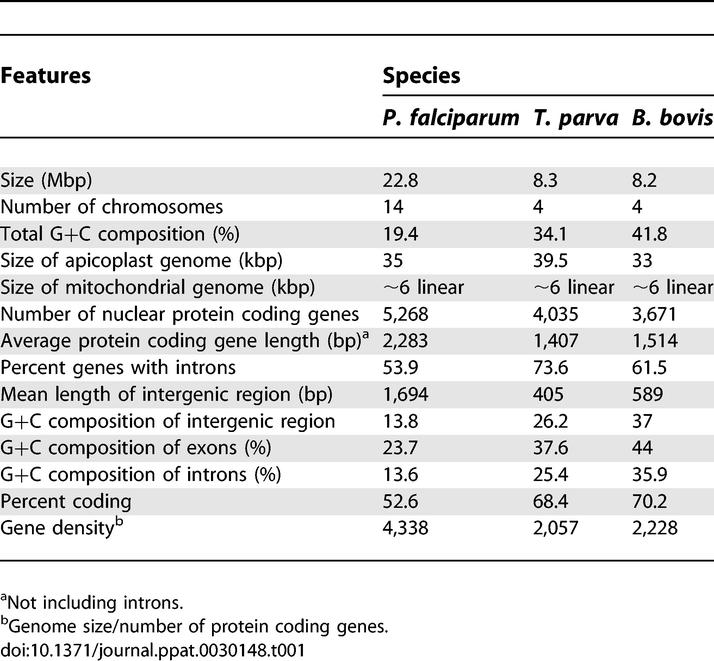
Genome Characteristics of B. bovis, T. parva, and P. falciparum

**Figure 1:**
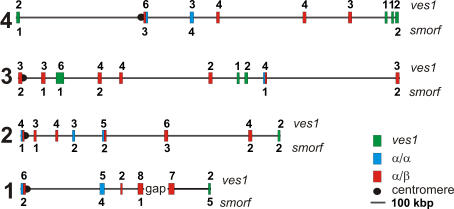
Representation of B. bovis Centromeres, *ves1*, and *smorf* Genes Each chromosome is represented by a black line, with the chromosome number shown on the left. Centromeres are depicted as black dots. *ves1* loci are depicted as colored boxes, and the number of *ves1* and *smorf* genes in each locus is shown above or below the colored boxes, respectively. Red and blue boxes indicate the presence of at least one *ves1α*/*ves1β* or *ves1α*/*ves1α* pair, respectively, within the cluster.

Each B. bovis chromosome contains an A+T-rich region ∼3 kbp in length presumed to be the centromere (Figure 1) based on features similar to those described for the putative centromere on P. falciparum chromosome 3 [14]. Three of the chromosomes are acrocentric, while chromosome 4 is submetacentric. The organization of telomeres and sub-telomeric regions resembles that seen in *Theileria* [7,10], as protein coding genes are found within 2–3 kbp of the end of CCCTA_3–4_ telomeric repeat sequences. The B. bovis genome contains three rRNA operons, two on chromosome 3 and one on chromosome 4, and 44 tRNA genes distributed across all four chromosomes. A total of 3,671 nuclear protein coding genes are predicted in the B. bovis assembled sequence data. In addition to the nuclear genome, the parasite contains two A+T-rich extra-chromosomal genomes: a circular 33 kbp apicoplast genome and a linear ∼6 kbp mitochondrial genome (Table 1), described below.

### Metabolic Potential and Membrane Transporters

**Figure 2:**
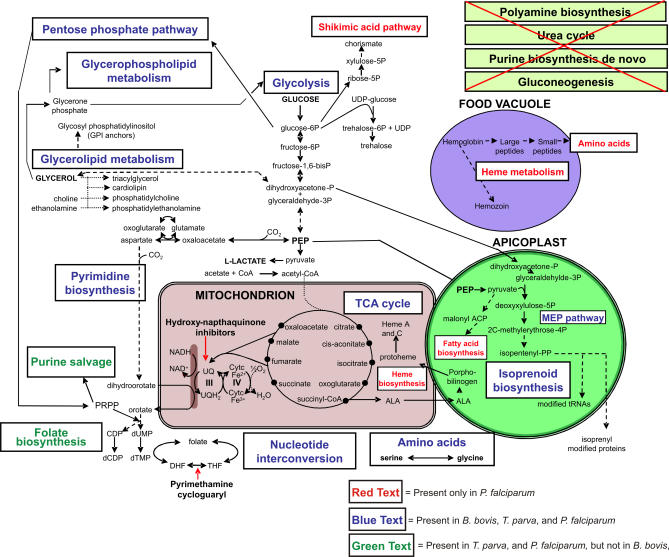
Comparison of Major Metabolic Pathways in B. bovis, T. parva, and P. falciparum Solid arrows indicate single step enzymatic reactions, dashed lines indicate multi-step reactions, and dotted lines indicate incomplete or unknown pathways. Inhibitory drugs are indicated with red arrows. Glucose is assumed to be the major carbon and energy source. Non-functional pathways in P. falciparum, T. parva, and B. bovis are shown in boxes with a red X. Pathways denoted with blue text are present in P. falciparum, T. parva, and B. bovis. Pathways denoted with green text are present in P. falciparum and T. parva but not in B. bovis, and pathways denoted with red text are exclusively found in P. falciparum.

A series of *in silico* metabolic pathways for B. bovis were reconstructed from 248 proteins assigned an EC number, including glycolysis, the tricarboxylic acid cycle and oxidative phosphorylation, de novo pyrimidine biosynthesis, glycerolipid and glycerophospholipid metabolism, the pentose phosphate pathway, and nucleotide interconversion (Figure 2). Notably, a number of major pathways appear to be lacking in the parasite, including gluconeogenesis, shikimic acid synthesis, fatty acid oxidation, the urea cycle, purine base salvage and folate, polyamine, type II fatty acid, and de novo purine, heme, and amino acid biosyntheses. Although heme biosynthesis activity present in P. falciparum is predicted to be absent in B. bovis, it does encode delta-aminolevulinic acid dehydratase (BBOV_II001120), which catalyzes the second step in heme biosynthesis.

The predicted metabolic profile of B. bovis is more similar to that of *Theileria* [7,10] than to that of P. falciparum [11]. Like *Theileria*, B. bovis does not appear to encode pyruvate dehydrogenase. Thus, there is no classical link between glycolysis and the tricarboxylic acid cycle. Interestingly, massively parallel signature sequencing has demonstrated that lactate dehydrogenase is the third most highly transcribed gene in T. parva schizonts [15], suggesting that in these organisms lactate may be the primary end product of glycolysis. This could be true for B. bovis as well. The enzymes adenine phosphoribosyltransferase and hypoxanthine-guanine phosphoribosyl-transferase (HGPRT) involved in salvage of purine bases appear to be lacking in *B. bovis*. HGPRT is present in P. falciparum (PF10_0121) [11], but absent from T. parva and T. annulata. Interestingly, although the purine salvage pathway is incomplete, B. bovis may be able to salvage purine nucleosides [16]. A recent analysis of B. bovis expressed sequence tags (ESTs) identified two adenosine kinases [17], a finding corroborated by the genome sequence data, which also revealed the presence of adenosine deaminase. These enzymes are absent in T. parva, while P. falciparum encodes adenosine deaminase. While we cannot exclude that HGPRT is present in the chromosome 1 gap, the apparent absence of HGPRT in B. bovis is in contrast to previous studies demonstrating the incorporation of radio-labeled hypoxanthine in parasite erythrocyte cultures [16,18]. Although several enzymes involved in purine salvage are present, there appears to be no direct path to the production of inosine monophosphate, and it is possible that the necessary enzymes are present but are not similar to known enzymes. Unlike P. falciparum and the Theileria spp., B. bovis does not appear to encode dihydrofolate synthase, which converts dihydropteroate to dihydrofolate. However, this deficiency could be compensated through importation via a folate/biopterin transporter (BBOV_IV002460) and increased dihydrofolate reductase–thymidylate synthase (DHFR-TS) activity. Consistent with a previous study using the Israel strain of B. bovis [19], the T2Bo DHFR-TS contains three of the four amino acid substitutions found in a mutant P. falciparum DHFR-TS with strong resistance to pyrimethamine, a DHFR inhibitor. An additional single point mutation is linked with the ability of B. bovis to develop strong resistance to pyrimethamine [19].

Babesia bovis has the smallest number of predicted membrane transporters [20] among the sequenced apicomplexan species (Table S1), but encodes more members of some families (for example, glucose-6-phosphate/phosphate and phosphate/phosphoenolpyruvate translocators, members of the drug/metabolite transporter superfamily). It encodes fewer members of the ABC efflux protein family than T. parva but has more transporters for inorganic cations, including a cation diffusion facilitator family protein that is absent in T. parva and other apicomplexans. Both B. bovis and T. parva lack aquaporins, the calcium:cation antiporters, and amino acid permeases that are present in the genome of P. falciparum. Orthologs of the different types of amino acid transporters cannot be identified in B. bovis, including the dicarboxylate/amino acid:cation (Na^+^ or H^+^) symporter family amino acid:cation symporter that is present in T. parva [10].

### The Apicoplast

Most members of the phylum *Apicomplexa* harbor a semi-autonomous plastid-like organelle termed the apicoplast, which was derived via a secondary endosymbiotic event [21]. The B. bovis apicoplast genome is 33 kbp and unidirectionally encodes 32 putative protein coding genes, a complete set of tRNA genes (25), and a small and large subunit rRNA gene (Figure S1). The B. bovis apicoplast genome displays similarities in size, gene content, and order to those of Eimeria tenella, P. falciparum, T. parva, and Toxoplasma gondii (Table S2; [22–24]). As observed with other apicoplast genomes, the B. bovis apicoplast genome is extremely A+T rich (78.2%), in contrast to the nuclear genome (58.2%).

In addition to the apicoplast genome encoded proteins, it has been demonstrated in P. falciparum that proteins encoded by nuclear genes are imported into the apicoplast (reviewed in [25]) to carry out a variety of metabolic processes, including heme biosynthesis [26], fatty acid biosynthesis [27], and isoprenoid precursor synthesis via the methylerythrose phosphate pathway [28]. Nuclear encoded proteins targeted to the apicoplast of P. falciparum have a bipartite targeting sequence consisting of a signal peptide that directs the protein to the secretory pathway and an apicoplast transit peptide that redirects the protein from the default secretory pathway into the lumen of the apicoplast [29,30].

Analysis of the metabolic functions ascribed to the apicoplast in P. falciparum reveals that only the enzymes for isoprenoid biosynthesis are found in B. bovis. To detect additional apicoplast-targeted proteins, PlasmoAP, a program developed to predict apicoplast targeting for P. falciparum [31], was used and revealed only 14 additional candidate proteins. This result is, perhaps, not unexpected, as the program was trained with P. falciparum sequences and likely works well only for P. falciparum because of skewed codon usage resulting from the low G+C content of P. falciparum. A third approach included visual inspection of BLAST search outputs of the entire B. bovis proteome against the nr database (National Center for Biotechnology Information) for potential amino-terminal extensions. This search resulted in 25 potential apicoplast-targeted sequences that had non-apicomplexan homologs with significant E values and bona fide amino terminal extensions. In total, 47 proteins (the eight involved in the methylerythrose phosphate pathway, 14 SignalP sequences identified with PlasmoAP, and 25 proteins identified through BLAST and visual inspection for amino terminal extentions) are predicted to be targeted to the B. bovis apicoplast (Table S3), by far the fewest of any organism for which this type of analysis has been done. P. falciparum and T. parva are predicted to have 466 and 345 apicoplast-targeted proteins, respectively [10,32]. The paucity of proteins predicted to be targeted to the B. bovis apicoplast may partially reflect the biology of the organism, with fewer functions attributed to the B. bovis apicoplast compared to P. falciparum, but is more likely a reflection of the lack of appropriate prediction algorithms.

The apicoplast has been an attractive target for development of parasiticidal drug therapies as the biosynthetic pathways represented therein are of cyanobacterial origin and differ substantially from corresponding pathways in the mammalian host [21,33]. A recent study of the apicomplexan T. gondii demonstrated that fatty acid synthesis in the apicoplast is necessary for apicoplast biogenesis and maintenance, and indicates that this pathway would be an ideal target for drug design [34]. Thus, the reduced metabolic potential of B. bovis has important ramifications for drug design, suggesting that drugs targeting fatty acid synthesis would not be effective against babesiosis due to the absence of this pathway.

### The Mitochondrion

B. bovis contains a 6 kbp linear mitochondrial genome (Figure S2). It encodes three putative protein coding genes, including cytochrome c oxidase subunit I, III, and cytochrome b. These are membrane-bound proteins that form part of the enzyme complexes involved in the mitochondrial respiratory chain. Cytochrome b and c subunit III are encoded on the same strand, while cytochrome c subunit I is encoded on the opposite strand. This coding arrangement is identical to that of Theileria spp. but different from that of P. falciparum [7,11,35]. Each of the encoded proteins employs the universal ATG as the start codon, in contrast to the T. parva cytochrome c subunit I, which has an AGT start codon [35]. In addition to the three protein coding genes, the B. bovis mitochondrial genome includes at least five partial rRNA gene sequences ranging in size from 34 to 301 bp. All five rRNA sequences are homologous to parts of the large ribosomal subunit of rRNA. They are encoded on both strands of the mitochondrial genome with rRNA 1 and 5 on the same strand and 2, 3, and 4 on the opposite strand. A terminal inverted repeat was identified from position 11–180 and 6005–5836.

### Protein Families

The B. bovis proteome was used to construct protein families using Tribe-MCL, a sequence similarity matrix-based Markov clustering method, and a method based on a combination of hidden Markov model domain composition and sequence similarity [36]. In addition to housekeeping gene families found in most eukaryotes, the pathogen contains only two large gene families. One of these families, encoding the variant erythrocyte surface antigen (VESA), has been previously defined [37]. The second, which we have termed SmORF (small open reading frame), is novel. Smaller notable families encode a 225 kD protein, known as spherical body protein 2 (SBP2) [38], and the variable merozoite surface antigen (VMSA) family [39].

#### VESA1.

**Figure 3:**
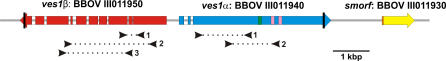
Diagram of a Locus Containing the *ves1α*, *ves1β*, and *smorf* Genes The genome backbone is a gray line, *ves1α* exons are blue, *ves1β* exons are red, and the SmORF exon is yellow. Introns are shown as blank boxes through which the genome backbone is seen. The systematic gene name for each gene is shown. Transmembrane helices (black bars), coiled-coil domains (green boxes), variant domains with conserved sequences 1 and 2 (pink boxes), and the SmORF signal peptide (orange box) are indicated. Arrows represent the positions of the primers for each of the cDNA experiments. The experiment number is indicated to the right of the primer sets, with experiment 1 targeting specific genes, experiment 2 sets of genes, and experiment 3 the published LAT.

VESA1 is a large (>300 kD), heterodimeric protein composed of VESA1a and VESA1b that is synthesized by B. bovis and subsequently exported to the surface of the host erythrocyte [40]. VESA1 undergoes rapid antigenic variation and has been implicated in host immune evasion and cytoadhesion, both of which would be expected to play a vital role in persistence and pathogenesis [41,42]. VESA1 is thought to be the functional homolog of PfEMP1, encoded by the *var* gene family, in P. falciparum [43]. The *ves1* genes comprise the largest family in the B. bovis genome. While sequence identity and the presence of similar secondary amino acid structures make it clear that these genes belong to the same family, two distinct types exist (*ves1α* and *ves1β*, encoding VESA1a and VESA1b, respectively) that possess highly variable regions of sequence composition, length, and gene architecture (Figure 3). Genomic analysis predicts 119 *ves1* genes in the available sequence (72α, 43β, and four unclassified; Table S4). However, there is a gradient of increasing concentration of *ves1* genes in the sequence immediately adjacent to the physical gap on chromosome 1, and the contigs that appear to reside in the gap contain *ves1*-like sequences, indicating that additional *ves1* genes reside in the gap. An estimated gap size of 150 kbp would limit the number of genes within the missing sequence to less than 40, resulting in a total of approximately 150 *ves1* genes, far fewer than previously predicted [37]. All but three members of the *ves1* family are found in clusters of two or more genes, with individual clusters separated by a few kilobases to nearly one megabase. Interestingly, *ves1* genes are distributed throughout all four chromosomes (Figure 1), in contrast to the observation that genes involved in antigenic variation, immune evasion, and sequestration, including *P. falciparum var* genes, are only occasionally found internally and are predominately telomerically located [11,44]. While *ves1* genes are also found near telomeres and centromeres, 89 genes (75%) are located distal to these chromosomal structures.

Transcription of *ves1* genes has been hypothesized to occur at a “locus for active transcription” (LAT), described as a divergently oriented pairing of *ves1α* and *ves1β* genes [37]. This large locus encompasses nearly 13 kbp and includes *ves1α* and *ves1β* (each >4 kbp), a short intergenic region (<500 bp), and short portions of each gene found as blocks of repeats and motifs downstream from each *ves1* coding region. The genome sequence contains 24 loci (Figure S3) with paired *ves1α*/*ves1β* genes with similar length, structure, and physical arrangement as found in the published LAT. This head-to-head arrangement is also found for 18 *ves1α* genes of similar length, resulting in nine loci containing *ves1α*/*ves1α* paired genes. These two groups of paired genes account for greater than half (66/119) of the annotated *ves1* genes (Table S4), and exhibit the highest level of sequence identity and structural similarity among *ves1α* and *ves1β* genes.

The remaining *ves1* genes cannot be easily sorted according to the previously described head-to-head arrangements, and many of these genes are significantly truncated. All of them can be classified as either *ves1α* or *ves1β*, with the exception of four *ves1* genes located on chromosome 3. It is possible that the genes not arranged in putative LATs represent ancestral forms of *ves1* and now play the role of functional pseudogenes, providing material for segmental gene conversion into a functional LAT to create antigenic variation [45].

*ves1α* and *ves1β* exhibit sequence similarity, but have different gene architecture. *ves1α* genes that are members of potential LATs tend to have three exons: two small exons followed by a large third exon are separated by two short introns. The *ves1β* genes show considerably more diversity, as they have numerous introns that are not consistent in length or location [37,41]. Even among *ves1β* genes in potential LATs, gene length varies from 987 to 3,642 bp, and the number of introns ranges between 2 and 11.

Although the *ves1α* and *ves1β* genes are structurally distinct, areas of sequence conservation and topological similarities exist among the predicted polypeptides. The corresponding conserved stretches of nucleotide identity may be exploited as recombination sites for the generation of antigenic diversity, in addition to encoding a functional motif. Because VESA1 is exported to the surface of infected erythrocytes [40], it is notable that only seven of the 119 potential products are predicted to possess an N-terminal signal peptide (which again suggests that current signal prediction algorithms may not be suitable for B. bovis). Most predicted VESA1 proteins have a large extracellular domain followed by a single transmembrane segment and a short cytoplasmic tail. This topology is conserved in VESA1a proteins encoded by genes in *ves1α*/*ves1β* and *ves1α*/*ves1α* pairings (35/42 *ves1α* genes), and to a lesser extent in VESA1b proteins encoded by genes in the *ves1α*/*ves1β* pairings (15/24 *ves1β* members). As with exon structure and gene length, however, considerably less conservation exists among the remaining proteins, as only 21/53 follow this pattern (Table S4).

VESA1a is distinguished from VESA1b by the presence of a coiled-coil domain located near the center of the predicted protein, with 83% of all VESA1a subunits and 98% of VESA1a subunits from potential LATs containing this domain. Of the 11 VESA1a subunits that do not contain the coiled-coil domain, eight are encoded by truncated genes containing less than 312 amino acids and none are encoded by genes exhibiting the typical three exon structure. In contrast to VESA1a, only 4/43 VESA1b subunits contain the coiled-coil domain. An additional characteristic found almost exclusively among the VESA1a subunits is the presence of two distinct motifs that are variable among the predicted protein sequences but contain invariant amino acids at specific positions. These domains, referred to as the variant domain conserved sequences one and two (VDCS-1 and −2) [37], are arranged in tandem and located near the coiled-coil domain. The T2Bo consensus sequence for VDCS-1 is K(N,D)x(L,I,V) (S,K)xxIxxxxxx(L,V) and for VDCS-2 is CxxCxxHxxKCGxxxxxxxCxxCx(Q,N)xxxxGXPS. While VESA1b subunits are essentially devoid of this motif, the VDCS-1 and −2 also help to define the subsets into which the VESA1a sequences are organized. VESA1a subunits predicted from the *ves1α*/*ves1β* pairs all possess perfect matches to VDCS-1 and −2 and only four motifs (three VDCS-1 and one VDCS-2) are missing from those coded by *ves1α*/*ves1α* pairs. In areas where these four missing motifs would normally be found, a similar amino acid pattern exists that does not match the motif perfectly. Of the remaining VESA1a subunits, 16/30 contain VDCS-1 and 17/30 possess VDCS-2.

Due to their resemblance to the published LAT [37], 33 pairs of *ves1* genes should be considered potential transcription sites. The potential LATs are not clustered, and are distributed throughout the chromosomes. To better understand whether one or more of these potential sites of transcription were active, we examined T2Bo *ves1* gene cDNA sequences. Primer sets for three different experiments were designed to target (1) specific genes, (2) sets of genes, or (3) the published LAT (Figure 3), and a total of 66 *ves1α* and 93 *ves1β* cDNA clones were analyzed. Unexpectedly, these cDNA represented 50 and 59 unique *ves1α* or *ves1β* sequences, respectively. Equally surprising, only one of the *ves1α* and none of the *ves1β* unique cDNA sequences matched a genomic *ves1α* or *ves1β* sequence. The *ves1* cDNAs displayed up to 50% sequence divergence in pairwise comparisons for transcripts within a given experiment. In experiment 1 (designed to target specific genes), 83% of the *ves1α* had >91% sequence identity in pairwise comparisons while the *ves1β* cDNAs displayed a bimodal distribution, with 46% having >91% sequence identity and 50% having only 56%–70% sequence identity. The RNA used for these experiments was obtained from B. bovis T2Bo culture more than two years following isolation of the genomic DNA used to construct the libraries used for sequencing, possibly accounting for some of the sequence diversity, i.e., due to changes in the population represented in the culture at the time of sampling. However, although variation over time may account for some of the differences between the cDNA and genomic sequences, the number of unique sequences obtained from a single time point exceeds the number of predicted expression sites for *ves1* genes. Consistent with this finding, numerous *ves1* genes were also represented in EST data [17]. *Var* gene expression, while “leaky” in the ring stages of P. falciparum, appears to be restricted to a single, or very few, alleles in individual parasite populations in vivo [46,47]. However, multiple *var* transcripts, although far fewer than for B. bovis, have been detected when the organism is cultured in vitro [48]. One possible explanation for the large number of different *ves1* cDNA transcripts may be that, similar to the *var* genes, *ves1* genes are removed from in vivo transcriptional controls and/or phenotypic selection when the organism is grown in vitro. While in vivo analysis of *ves1* transcription remains to be performed, the number of diverse transcripts is interesting, and may suggest more widespread transcription and alternative post-transcriptional control mechanisms than observed in other hemoprotozoa.

#### SmORFs.

Found associated with the *ves1* genes across all four chromosomes are members of the second largest protein family (SmORFs) in the B. bovis genome (Figures 1 and 2). These “small open reading frames” (so named due to their association with, but smaller size than the *ves1* genes) include 44 genes with lengths ranging from 381 to 1,377 nucleotides with no significant sequence identity to any protein or gene sequence in available databases. When compared to VESA proteins, a higher degree of sequence conservation (∼50% amino acid identity for most pairwise comparisons, with a range from 28%–95%) is found among SmORF paralogs, and 42/44 members consist of a single exon. Additionally, 43 family members are predicted to have a signal peptide, and all 44 are predicted by TMHMM [49] to exist extracellularly. Alignment of the SmORF sequences reveals four blocks of conserved sequence interrupted by linking sequence present in only one or a few of the SmORF paralogs (Figure S4). These long linking sequences interspersed between the blocks of identity in a few proteins account for the increased peptide length for the longer members of the family. Results from experiments designed to detect *smorf* transcripts were similar to that for *ves1*: two of five cloned products matched the predicted genome sequence while the remaining clones differed. The prevalence of canonical signal peptides among SmORFs, and their uniform association with *ves1* clusters, tempts speculation that these proteins may play a functional role in VESA1 biology, or may, themselves, contribute to antigenic variation and immune evasion, or both. However, elucidation of the function of these proteins awaits biochemical and immunological analysis.

#### SBP2 family.

The spherical body is an apical organelle thought to be analogous to dense granules in other apicomplexan organisms [50]. SBP2 (also known as BvVa1) is a 225 kD immunostimulatory protein from the spherical body that is released into the host erythrocyte upon invasion and localizes to the cytoplasmic side of the erythrocyte membrane [38,51–53]. The original study noted that there were multiple copies of the 5′ end of the gene, while the 3′ end appeared to be single copy [51]. Consistent with this study, the genome sequence reveals that there are 12 truncated copies of the SBP2 gene corresponding to the 5′ end of the full-length gene, and one full-length copy. The full-length gene and one truncated gene are on chromosome 4, with all remaining truncated copies on chromosome 3. The truncated genes on chromosome 3 occur in three clusters of two, four, and five genes. The genes occurring in the 2- and 5-gene clusters are interspersed with another set of highly conserved (88%–100%) gene repeats (BBOV_III005620, BBOV_III006470, BBOV_III006490, BBOV_III006510, and BBOV_III006530) that have no homologs in the public databases. The 12 truncated SBP2 genes have sequence identities ranging from 27%–99% in pairwise comparisons, with the greatest identity in the first 30 amino acids. Previous analysis of EST sequences indicates that more than one of these truncated genes are transcribed [17].

#### VMSA.

The variable merozoite surface antigen genes encode a family of immunostimulatory proteins that are a target of invasion blocking antibodies [39,54,55]. As in related Mexico strains of B. bovis [56], the T2Bo genome contains five *vmsa* genes, including *msa1* and four copies of *msa2*. The VMSA genes reside on chromosome 1, with the four *msa2* copies arranged tandemly in a head-to-tail fashion, and *msa1* residing ∼5 kbp upstream from the *msa2* genes. Interestingly, the VMSAs do not have homologs in Theileria spp. or P. falciparum.

### Comparative Analyses of Hemoprotozoan Proteomes

#### Clusters of orthologous groups.

Similarity clustering using the predicted proteomes of B. bovis, T. parva, and P. falciparum created 1,945 three-way clusters of orthologous groups (COGs) (Figure S5; Table S5). As expected from phylogenetic studies, the B. bovis proteome is more closely related to that of T. parva. Approximately half of the remaining B. bovis proteins not included in the three-way COGs fell into two-way COGs with proteins from T. parva, while B. bovis and P. falciparum shared only 111 two-way COGs. Remaining after cluster analysis were 706, 1,107, and 3,309 unique genes for B. bovis, T. parva, and P. falciparum, respectively (Tables S6–S8).

Since P. falciparum, T. parva, and B. bovis all have complex life cycles that involve arthropod vector and mammalian host stages, Jaccard-filtered COG (Jf-COG) data were used to search for B. bovis orthologs of proteins that have been characterized in T. parva and P. falciparum as targets of protective immune responses, as well as those that play a role in stage-specific parasite biology. Many genes exclusively expressed in sexual stages of P. falciparum (for example PfCDPK3, PfLAMMER, and Pfs230) do not share Jf-COGs with T. parva or B. bovis, a difference potentially associated with the different vector (mosquito versus tick) that transmits *Plasmodium*. Likewise, P. falciparum sporozoite genes that are expressed initially in the mosquito, such as Pfs 25/28, exported protein 2, circumsporozoite protein, circumsporozoite protein/thrombospondin-related anonymous protein-related protein, and sporozoite microneme protein essential for cell traversal 1 and 2, also do not cluster with T. parva or B. bovis proteins*.* Since B. bovis does not have a pre-erythrocytic liver stage, as expected, orthologs of P. falciparum liver stage proteins such as PfLSA 1–3 are not detected. P. falciparum erythrocytic stage proteins such as the PfMSPs were not detected in B. bovis, nor were plasmodial rhoptry and rhoptry-associated proteins (RAPs). However, BbRAP-1a (BBOV_IV009860 and BBOV_IV009870) forms a Jf-COG with its T. parva (TP01_0701) and T. annulata (TA05760) orthologs. Interestingly, B. bovis encodes a protein (RRA; BBOV_IV010280) most similar to RAP-1b, previously described only in B. bigemina [57].

Noteworthy P. falciparum genes that have Jf-COGs with B. bovis are thrombospondin-related anonymous protein (TRAP), p36 protein, Pf12, Sir2, PfATP6, and P0. PfTRAP is expressed exclusively in sporozoites, while BbTRAP is expressed in both sporozoite and blood stages [58]. A plasmodial surface membrane protein, p36 is a member of the p45/48 sporozoite protein family. It participates in liver stage parasite development, and immunization with Pfp36 knockout parasites results in protective immunity against subsequent challenge with wild-type sporozoites, identifying p36 as a potential knockout gene for development of attenuated vaccines [59]. Pf12 is a merozoite surface protein that is recognized strongly by antibodies of naturally infected individuals [60]. An ortholog of the Sir2 protein, involved in *P. falciparum var* gene silencing [61], is present in B. bovis (BBOV_I003070), forming a Jf-COG with orthologs in P. falciparum (PF13_0152), T. parva (TP01_0527), and Cryptosporidium parvum (cgd7_2030), but is apparently absent from T. annulata. An ortholog of PfATP6, the gene thought to be the target of the drug artemisinin used to treat drug-resistant malaria, is found in B. bovis (BBOV_II005700) [62]. Finally, BBOV_IV004540 forms a Jf-COG with P0 from P. falciparum (PF11_0313), T. parva (TP01_0294), and T. annulata (TA21355). P0 is a ribosomal phosphoprotein with immunoprotective properties [63].

Immunostimulatory proteins that form Jf-COGs with B. bovis include a T. parva protein annotated as polymorphic immunodominant protein (PIM) (TP04_0051). This polymorphic immunodominant protein is the target of sporozoite neutralizing antibodies [64], and falls into a Jf-COG with B. bovis protein BBOV_II005100, T. annulata protein TA17315, known as TaSP [65], and P. falciparum protein PF14_0369. However, the orthologs are half the length of the T. parva protein and do not contain a Q/E-rich central repeat domain that is characteristic of PIM. Of six additional antigens from T. parva (TP01_0056, TP02_0849, TP02_0767, TP02_0244, TP02_0140, TP03_0210) that are the targets of parasite-specific bovine MHC class I–restricted CD8^+^ cytotoxic T cells [66], four have orthologs in B. bovis (BBOV_IV000410, BBOV_IV006970, BBOV_III011550, BBOV_III004230, BBOV_III010070) and P. falciparum (PFC0350c, PF13_0125, MAL7P1.14, PF11_0447, PF14_0417, PF07_0029). BBOV_IV000410, one of the genes not found in P. falciparum, encodes a signal peptide-containing protein whose T. annulata homolog is targeted to the membrane [67]. B. bovis ACS-1 (BBOV_III010400) has been shown to stimulate CD4^+^ T lymphocyte responses in immune cattle [68], and forms a Jf-COG with T. parva (TP02_0107) and P. falciparum (PFL1880w) proteins. The B. bovis apical membrane antigen 1 (AMA-1; BBOV_IV011230) [69] is a micronemal protein that forms a Jf-COG with P. falciparum (PF11_0344), T. parva (TP01_0650), and T. annulata (TA02980) and has additional homologs with other apicomplexans. The B. bovis AMA-1 gene is located on chromosome 4 and is part of a syntenic cluster of four genes present across the P. falciparum, T. parva, and T. annulata genomes.

A unique aspect of T. parva and T. annulata is the ability of the schizont stage of these parasites to transform the leukocytes they reside in to a cancer-like phenotype [70]. This reversible change is dependent on the presence of viable parasites. Although a number of *Theileria* molecules that could interfere with host cell signaling pathways controlling cell proliferation and apoptosis have been mined from the genome sequence of both pathogens, no single molecule in either parasite could be linked with the phenotype. In general, both parasites encode the same repertoire of candidate proteins, suggesting that subtle differences account for the observation that T. parva transforms T and B cells while T. annulata transforms B cells and macrophages. As anticipated, the expansion in the number of genes coding for choline kinase in T. parva and T. annulata, which may contribute to increased lipid metabolism in transformed cells, is not present in B. bovis, which encodes a single copy of this gene. In an effort to further refine a list of candidate transformation-associated genes for T. parva, we analyzed a list of 1,107 T. parva proteins that do not fall into a Jf-COG with proteins from P. falciparum or B. bovis (Table S7). There are 262 proteins predicted to contain a signal peptide or signal anchor and are not predicted to be targeted to the apicoplast. Cross-referencing this list with transcriptional data derived from oligonucleotide based microarrays comparing T. parva schizonts and sporozoites reveals that 35 genes in the list are highly expressed in schizonts. These include two members of the TashAT gene family previously implicated in T. annulata transformation [71], and one member of a telomeric gene family [7]. It is notable that the remaining genes are all annotated as hypothetical proteins, emphasizing the need for a concerted effort to study the role of these novel proteins.

#### Syntenic analyses.

It is possible that due to evolutionary pressure, functional B. bovis homologs of T. parva and P. falciparum proteins may have diverged in sequence to the point they are no longer recognizable at the level of the primary amino acid sequence. For this reason, we examined the conservation of gene order in syntenic blocks between the pathogens. Syntenic blocks were defined as a pair of genes that belong to the same Jf-COG, where members of the pair belong to the reference and query sequence [10]. Even by this method, we were unable to identify obvious homologs for many P. falciparum proteins involved in stage-specific biology or host immunity. However, in T. parva, the regions flanking a gene encoding an abundant sporozoite surface antigen, p67, a primary target of parasite neutralizing antibodies [72], form a highly conserved syntenic block with B. bovis and T. annulata (Figure S6). Sporozoite antigen 1 (SPAG-1), the positional homolog of p67 in T. annulata, is itself known to contain neutralizing epitopes and is a leading vaccine candidate [73]. The gene in B. bovis (BBOV_IV007750) that occupies the site of p67 in T. parva is predicted to encode a membrane protein, suggesting that this protein may have immunostimulatory properties equivalent to p67. RT-PCR experiments indicate that the B. bovis gene is transcribed in infected erythrocytes and during the kinete stage in ticks (unpublished data; sporozoite expression has not been examined). It will be interesting to explore the vaccine potential of the B. bovis p67 homolog, as ∼50% of cattle immunized with recombinant p67 and challenged under field conditions show a reduction in severe East Coast fever [74].

**Figure 4:**
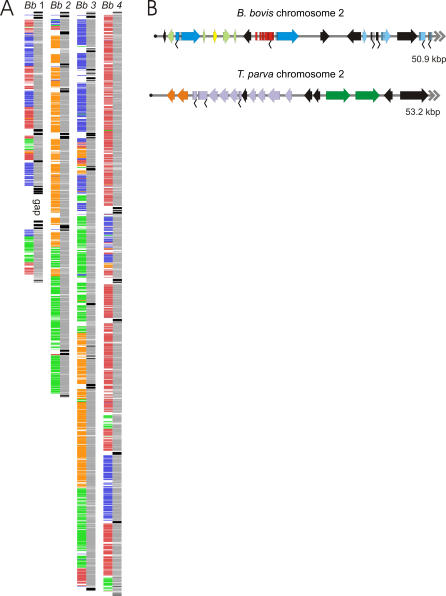
Diagram of Chromosomal Synteny between B. bovis and T. parva (A) Synteny at the chromosomal level. B. bovis chromosome number is indicated at the top. Bars on the right side of each chromosome diagram designate B. bovis genes, with black bars indicating *B. bovis ves1* genes and gray bars indicating other genes. The colors on the left of each chromosome diagram indicate to which T. parva chromosome an ortholog belongs as follows: Tp1 = red, Tp2 = green, Tp3 = blue, Tp4 = orange. (B) Comparison of telomeric arrangement of genes for B. bovis and T. parva chromosome 2. The gray line indicates the chromosomal backbone, with black dots indicating the telomere. Large genes are depicted as arrows with coding direction indicated, while small genes have an arrowhead beneath the gene to indicate the direction of transcription. Gray double arrowheads indicate that the chromosome continues. Colors indicate gene content as follows: blue = *B. bovis ves1α*, red = *B. bovis ves1β*, yellow = B. bovis SmORF, pale green = putative membrane protein, pale blue = annotated genes with predicted function, black = hypothetical, orange = T. parva family 3 hypothetical, purple = T. parva family 1 hypothetical, green = ABC transporter.

Large blocks of synteny are evident between B. bovis and T. parva chromosomes (Figure 4A). However, several chromosomal rearrangements have taken place, as observed between chromosomes of P. falciparum and *P. yoelii yoelii* [75]. Synteny rarely extends to telomeres (Figure 4B), as these regions usually contain species-specific polymorphic genes that are present at many syntenic break points. Unlike the T. parva subtelomeric regions, the B. bovis subtelomeres contain genes transcribed from both strands. However, similar to both T. parva and P. falciparum, the telomeres contain many (putative) membrane proteins. At a gross level, B. bovis chromosomes 2 and 4 primarily consist of sections of T. parva chromosome 4 and 2, and 3 and 1, respectively. B. bovis chromosome 3 contains sections from all four T. parva chromosomes, while B. bovis chromosome 1 contains DNA from T. parva chromosomes 3, 1, and 2 (Figure 4). Closer examination of syntenic blocks indicates that inversions in gene order have also taken place.

### Summary

The 8.2 Mbp genome of B. bovis consists of four nuclear chromosomes, and two small extra-nuclear chromosomes for the apicoplast and mitochondria. B. bovis appears to have one of the smallest apicomplexan genomes sequenced to date. Consistent with the small genome size, analysis of enzyme pathways reveals a reduced metabolic potential, and provides a better understanding of B. bovis metabolism and potential avenues for drug therapies. Using several different approaches, identification of proteins predicted to be targeted to the apicoplast reveals far fewer proteins than for related organisms. This may be due in part to the lack of appropriate detection algorithms. However, the conservative approach used to identify the genes encoding these proteins provides a solid base from which to extend these analyses. A foundation for the elucidation of antigenic variation and immune evasion has been established with genome-wide characterization of the *ves1* gene family, and discovery of the novel *smorf* gene family. *ves1* and *smorf* genes are co-distributed throughout the chromosome, with the majority located away from telomeres and centromeres. As many as 33 potential loci of *ves1* transcription have been identified, and cDNA analysis suggests that this transcription is more broad-based than with other hemoprotozoa. Comparative analysis indicates that many stage-specific and immunologically important genes from P. falciparum are absent in B. bovis. However, through both COG analysis and synteny, additional B. bovis vaccine candidates, including homologs of P. falciparum p36, Pf12, T. parva p67, and four of six T. parva proteins targeted by CD8^+^ cytotoxic T cells, have been identified.

## Methods

### Parasite culture and library construction.

R. microplus adults were allowed to feed on calf C-912 inoculated with the T2Bo strain that was one passage (splenectomized calf) removed from a field isolate and frozen as a liquid nitrogen stabilate [76]. Progeny larvae were placed on calf C-936, blood was collected 7 d post tick infestation, and microaerophilous stationary phase culture was established according to [77] with modifications as described in [18]. Parasite genomic DNA from parasites in culture for 34–39 weeks was extracted using standard methods [78]. Small (2–3 kbp) and medium (12–15 kbp) insert libraries were constructed by nebulization and cloning into pHOS2. A large insert library (100–145 kbp) was constructed in pECBAC1 (Amplicon Express) and consisted of clones resulting from HindIII or MboI partially digested DNA.

### Genome sequencing.

A total of 103,478 high quality sequence reads (average read length = 870) were generated (58,251 reads from the small insert library and 45,227 reads from the medium insert library) and assembled using Celera Assembler (http://sourceforge.net/projects/wgs-assembler/). The sequence data fell into 50 scaffolds consisting of 88 contigs. The bacterial artificial chromosome library was end sequenced to generate an additional 2,874 reads that were used to confirm the assembly and for targeted sequencing in the closure phase. Gaps in the assembly were closed by a combination of primer walking and transposon based or shotgun sequencing of medium insert clones, bacterial artificial chromosome clones, or PCR products. This genome project has been deposited at DDBJ/EMBL/GenBank under accession number AAXT00000000. The version described in this paper is the first version, AAXT01000000.

### Functional annotation.

Chromosomal gene models were predicted using Phat [79], GlimmerHMM, TigrScan [80], and Unveil [81] after training each gene finding algorithm on 499 partial and full-length B. bovis genes totaling ∼453 kbp. The training data were manually constructed after inspection of the alignment of highly conserved protein sequences from nraa using the AAT package [82] and PASA to align a collection of ∼11,000 B. bovis ESTs [17] to the genome sequence. Jigsaw was used to derive consensus gene models [83] from the outputs of the gene finding programs and protein alignments. The consensus gene models were visually inspected and obvious errors such as split or chimeric gene models were corrected based on either EST or protein alignment evidence using the Neomorphic Annotation Station [84] before promotion to working gene models. Genes encoding tRNAs were identified using tRNAscan-SE [85].

BLAST [86] was used to search nraa using the predicted B. bovis protein sequences, and protein domains were assigned using the InterPro database [87]. The presence of secretory signals and transmembrane domains were detected using SignalP [88] and TMHMM [49], respectively. Functional gene assignments were assigned based on the BLAST data, and a Web-based tool called Manatee (http://manatee.sourceforge.net/) was used to manually curate and annotate the data. Proteins were annotated as hypothetical proteins if there was less than 35% sequence identity to known proteins, and as conserved hypothetical proteins if there was greater than 35% sequence identity to other proteins in the database that were unnamed. If a protein was predicted to have a signal peptide and at least one transmembrane domain, but was otherwise considered as a hypothetical or conserved hypothetical protein, it was annotated as a membrane protein, putative. If there was greater than 35% sequence identity for 70% of the sequence length, the protein product would be assigned a name only when a publication record could verify the authenticity of the named product. In the absence of published evidence, the named product was listed as putative. The mitochondrial and apicoplast genomes were manually annotated, and apicoplast-targeted proteins were analyzed using PlasmoAP (http://v4-4.plasmodb.org/restricted/PlasmoAPcgi.shtml) [31]. PASA [89] was used to align ∼86% of the B. bovis ESTs to the genome sequence data and provided evidence for transcription of 1,633 genes.

### Comparative analyses.

Sybil (http://sybil.sourceforge.net/) was used to create an all-versus-all BLASTP search using the proteomes of B. bovis, T. parva, and P. falciparum. These outputs were subjected to Jaccard clustering [10], placing proteins into distinct clusters for each proteome. Clusters from different proteomes were linked based on best bidirectional BLASTP hits between them to provide Jf-COGs. A minimum block size of five with one gap was allowed in the analyses.

### cDNA analyses.

Analysis of *ves1* transcription utilized total RNA isolated from microaerophilous stationary phase culture culture using TRIzol (BRL) treated three times with RNase-free DNase (Ambion) for 30 min at 37 °C. RNA was reverse transcribed with a Superscript (Invitrogen) reverse transcription kit using random hexamers according to the manufacturer's instructions. Universal primer sequences that would anneal to the two specific subunit types could not be found. Therefore, in the first RT-PCR experiment, primers were designed to amplify as many of the genes as possible. The following primers were used for *ves1β* cDNA: beta2For: 5′ GGA CTA CAG AAG TGG GTT GGG TGG and beta4Rev: 5′ ATA GCC CAT GGC CGC CAT GAA TGA; *ves1α* cDNA: alpha3For: 5′ CAG GTA CTC AGT GCA CTC GTT GGG TGG AG and alpha6Rev: 5′ CCC TAA TGT AGT GNA CCA CCT GGT TGT ATG C. Due to the high degree of sequence similarity (>99%) of the published *ves1* loci in cosmid 53 and 54 (accession numbers AY279553 and AY279554, respectively) to the genome sequence, a second RT-PCR experiment used primers designed to amplify the published LAT [37]. This experiment used primers LATbetaF1: 5′ GCA ACC GCA CGA CAG and LATbetaR2: 5′ CGC TGA CAC GCT AGT for the *ves1β* gene. A final cDNA cloning experiment was designed to elucidate the transcriptional profile for *ves1* by targeting *ves1α* and *ves1β* genes associated with Rep sequence clusters [37]. Primers were as follows: *ves1β*: 00789F1: 5′ AGA CTG TGA ATC TCG GCT CA and 00789R: 5′ CAG CGG CAC CAC TAC CTT T; *ves1α*: 00792F2: 5′ TGC CCA GGA CAG TTA TG and 00792R2: 5′ TGA TGC CCT CTT CAA TAG TT. Whenever possible, *ves1* primers were designed such that they would flank introns, providing an additional assurance that the amplicon obtained was not from contaminating genomic DNA; however, this was only possible for *ves1β* experiments.

## Supporting Information

Figure S1 Diagram of B. bovis Apicoplast Genome Coding sequences (CDSs) with functional annotation are depicted in red, while hypothetical CDSs are shown in lack. Genes encoding tRNA genes are shown as blue bars, while rRNA genes are shown as yellow arrows. All genes are unidirectionally encoded.
(303 KB PDF)

Figure S2 Diagram of B. bovis Mitochondrial Genome CDSs are depicted in red, large subunit rRNA genes in blue, and inverted terminal repeats as black arrows.
(38 KB PDF)

Figure S3 Diagram of the 24 Putative *ves1* LAT Loci All loci are depicted with *ves1β* on the left and *ves1α* on the right and drawn to the same scale. The genome backbone is a yellow line, exons are orange, and introns are shown as blank boxes. The systematic gene name for each gene is shown. Transmembrane helices (TMHelix), coiled-coil domains (Coiled Coil), and the variant domains with conserved sequences 1 and 2 (VDCS-1 or −2) are indicated.
(327 KB PDF)

Figure S4 Alignment of 44 SmORF Sequences Deduced amino acid sequences were aligned with the AlignX module of VectoNTI. The blue background indicates positions with identical amino acid sequences, while the green background indicates conserved amino acids. Dashes indicate that there is no amino acid in that position. Long stretches of intervening amino acid sequence has been trimmed from a few sequences to allow visualization of the four blocks of amino acid conservation. The double slashes (//) indicate that the sequence was trimmed from this position that spanned the full length between the remaining amino acids, while a single slash (/) indicates that the sequence that was trimmed from the alignment that did not fully span the region was removed. The total alignment length is 720 amino acids in length.
(43 KB PDF)

Figure S5 Venn Diagram Showing Number of Genes in Overlapping COGs between B. bovis, T. parva, and P. falciparum
(14 KB PDF)

Figure S6 Example of Microsynteny between B. bovis and T. parva
The T. parva p67 locus is diagrammed in the middle row, with the corresponding B. bovis locus shown on the top row, and the T. annulata SPAG-1 locus diagrammed on the bottom row. Genes with sequence identity between species are connected by shaded gray lines. The gene highlighted with pink was used to identify the syntenic locus. The p67 gene is indicated.
(52 KB PDF)

Table S1 Transporters (85 KB DOC)

Table S2 Characteristics of Apicomplexan Plastids (34 KB DOC)

Table S3 Nuclear Encoded Genes Potentially Targeted to the Apicoplast (104 KB DOC)

Table S4 Characteristics of *ves1* Sequences (44 KB XLS)

Table S5 CDS common to B. bovis, T. parva, and P. falciparum
(728 KB XLS)

Table S6 CDS Unique to B. bovis
(97 KB XLS)

Table S7 CDS Unique to T. parva
(138 KB XLS)

Table S8 CDS Unique to P. falciparum
(388 KB XLS)

### Accession Number

The B. bovis T2Bo genome is deposited at DDBJ/EMBL/GenBank under accession number AAXT00000000.

## Abbreviations

COG: cluster of orthologous group
DHFR: dihydrofolate reductase
EST: expressed sequence tag
HGPRT: hypoxanthine-guanine phosphoribosyl-transferase
Jf-COG: Jaccard-filtered cluster of orthologous group
LAT: locus of active transcription
PIM: polymorphic immunodominant protein from T. parva
SBP2: spherical body protein 2
SmORF: small open reading frame
TRAP: thrombospondin-related anonymous protein
VDCS: variant domain conserved sequence
VESA: variant erythrocyte surface antigen protein
VMSA: variable merozoite surface antigen
