# Supplementary material for: Genome Sequence of Babesia bovis and Comparative Analysis of Apicomplexan Hemoprotozoa
Source: PLoS Pathog. 2007 Oct 19;3(10):e148. doi: 10.1371/journal.ppat.0030148 (PMC2034396; doi:10.1371/journal.ppat.0030148)
Supplement: Supplementary file 1 [file ppat.0030148.sg001.pdf]

Supplementary Figure 1:

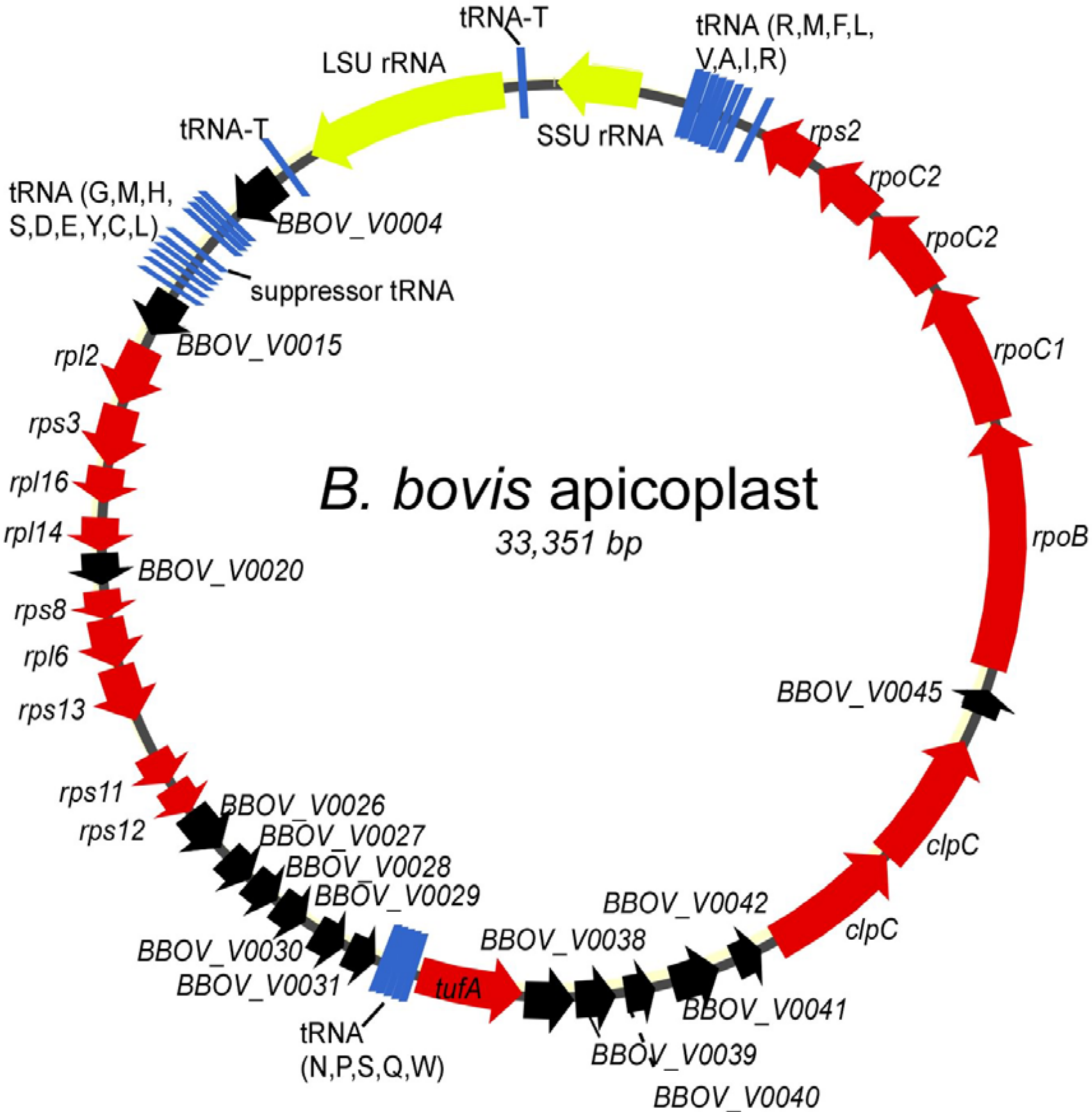

Supplementary Figure 1: Diagram of *B. bovis* apicoplast genome. CDSs with functional annotation are depicted in red, while hypothetical CDSs are shown in black. Genes encoding tRNA genes are shown as blue bars, while rRNA genes are shown as yellow arrows. All genes are unidirectionally encoded.
