# Supplementary material for: Genome Sequence of Babesia bovis and Comparative Analysis of Apicomplexan Hemoprotozoa
Source: PLoS Pathog. 2007 Oct 19;3(10):e148. doi: 10.1371/journal.ppat.0030148 (PMC2034396; doi:10.1371/journal.ppat.0030148)
Supplement: Supplementary file 2 [file ppat.0030148.sg002.pdf]

Supplementary Figure 2:

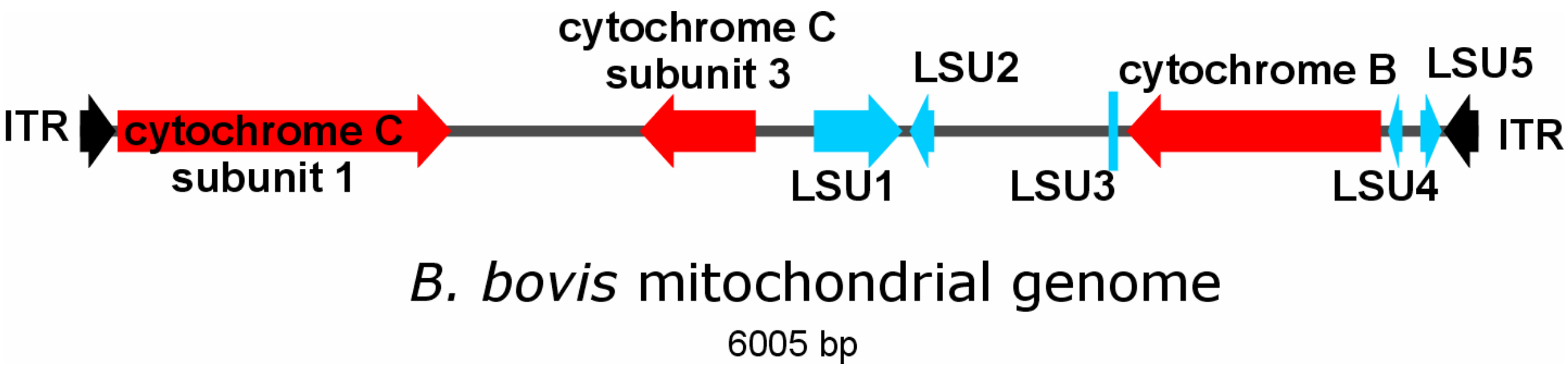

Supplementary Figure 2: Diagram of *B. bovis* mitochondrial genome. CDSs are depicted in red, large subunit rRNA genes in blue, and inverted terminal repeats as black arrows.
