# Supplementary material for: Genome Sequence of Babesia bovis and Comparative Analysis of Apicomplexan Hemoprotozoa
Source: PLoS Pathog. 2007 Oct 19;3(10):e148. doi: 10.1371/journal.ppat.0030148 (PMC2034396; doi:10.1371/journal.ppat.0030148)
Supplement: Supplementary file 3 [file ppat.0030148.sg003.pdf]

Supplementary Figure 3:

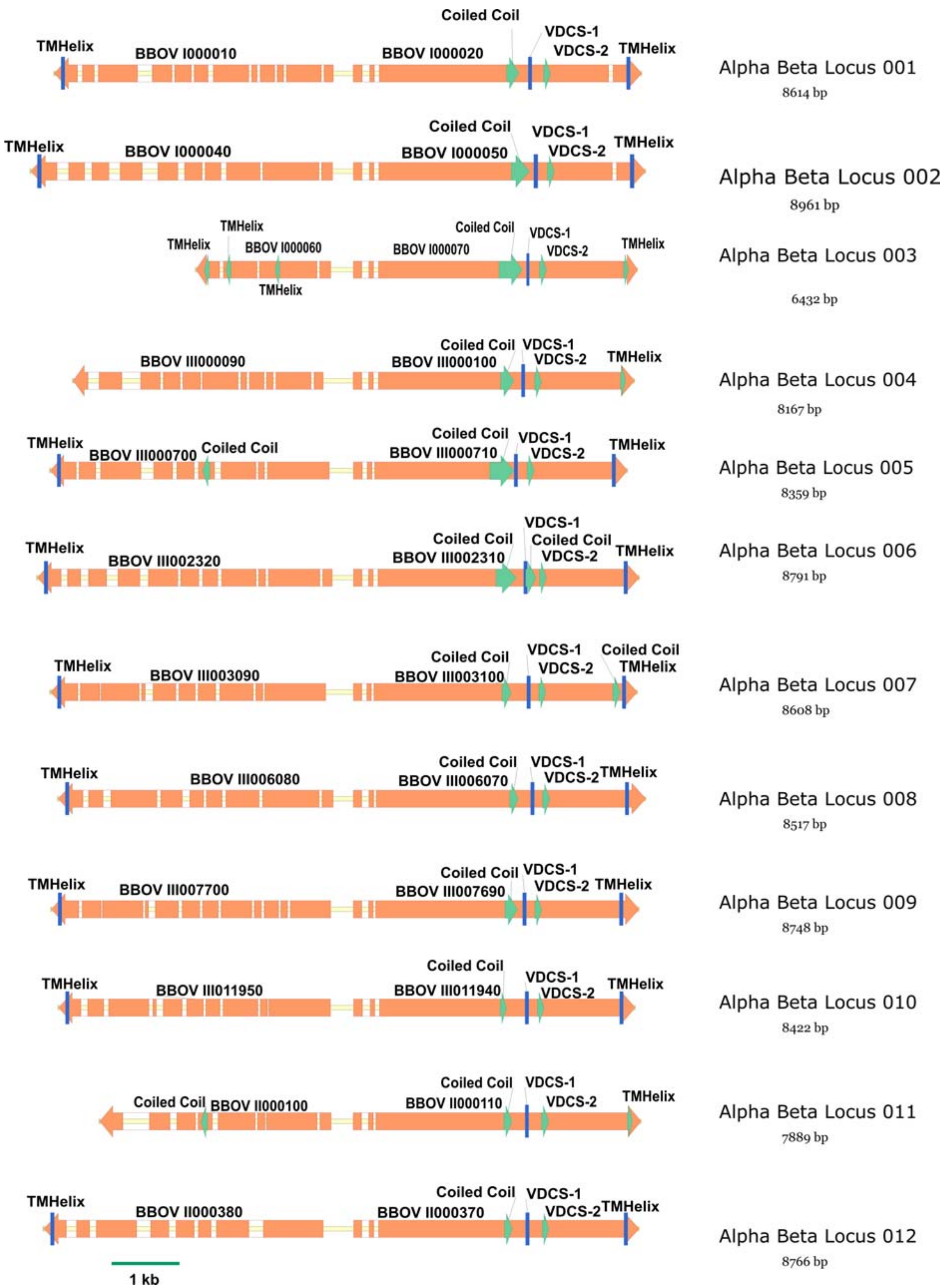

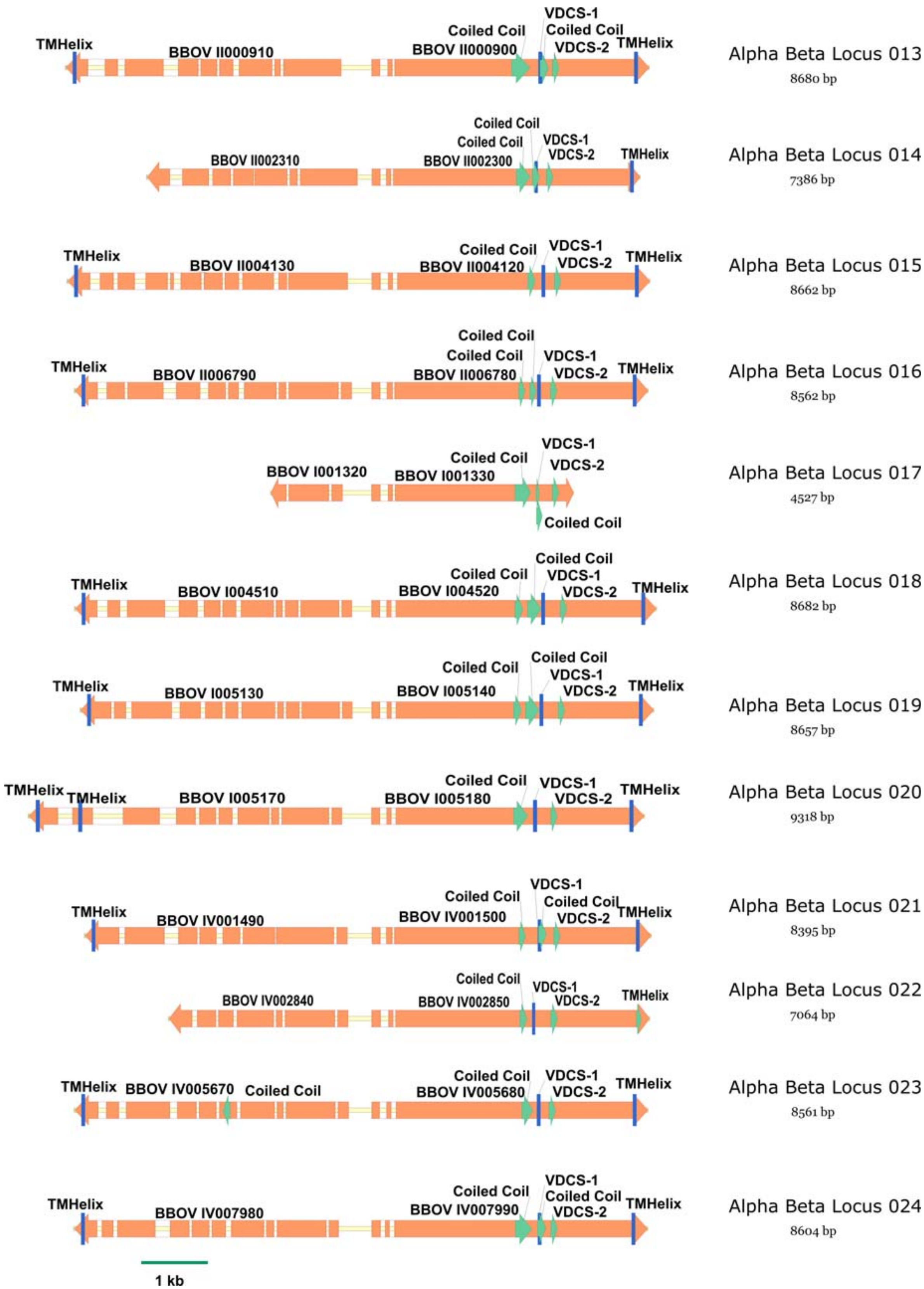

TMHelix
BBOV II000910
Coiled Coil
BBOV II000900
VDCS-1
Coiled Coil
VDCS-2
TMHelix
Alpha Beta Locus 013
8680 bp
Coiled Coil
Coiled Coil
BBOV II002310
BBOV II002300
VDCS-1
VDCS-2
TMHelix
Alpha Beta Locus 014
7386 bp
TMHelix
BBOV II004130
Coiled Coil
BBOV II004120
VDCS-1
VDCS-2
TMHelix
Alpha Beta Locus 015
8662 bp
Coiled Coil
Coiled Coil
TMHelix
BBOV II006790
BBOV II006780
VDCS-1
VDCS-2
TMHelix
Alpha Beta Locus 016
8562 bp
VDCS-1
Coiled Coil
VDCS-2
BBOV I001320
BBOV I001330
Coiled Coil
Alpha Beta Locus 017
4527 bp
Coiled Coil
Coiled Coil
VDCS-1
TMHelix
BBOV I004510
BBOV I004520
VDCS-2
TMHelix
Alpha Beta Locus 018
8682 bp
Coiled Coil
Coiled Coil
VDCS-1
TMHelix
BBOV I005130
BBOV I005140
VDCS-2
TMHelix
Alpha Beta Locus 019
8657 bp
Coiled Coil
VDCS-1
TMHelix
TMHelix
BBOV I005170
BBOV I005180
VDCS-2
TMHelix
Alpha Beta Locus 020
9318 bp
VDCS-1
Coiled Coil
Coiled Coil
TMHelix
BBOV IV001490
BBOV IV001500
VDCS-2
TMHelix
Alpha Beta Locus 021
8395 bp
Coiled Coil
VDCS-1
BBOV IV002840
BBOV IV002850
VDCS-2
TMHelix
Alpha Beta Locus 022
7064 bp
Coiled Coil
VDCS-1
TMHelix
BBOV IV005670
Coiled Coil
BBOV IV005680
VDCS-2
TMHelix
Alpha Beta Locus 023
8561 bp
VDCS-1
Coiled Coil
Coiled Coil
TMHelix
BBOV IV007980
BBOV IV007990
VDCS-2
TMHelix
Alpha Beta Locus 024
8604 bp
1 kb

Supplementary Figure 3: Diagram of the 24 putative *ves1* LAT loci. All loci are depicted with ves1β on the left and ves1α on the right and drawn to the same scale. The genome backbone is a yellow line, exons are orange, and introns are shown as blank boxes. The systematic gene name for each gene is shown. Transmembrane helices (TMHelix), coiled coil domains (Coiled Coil), and the variant domains with conserved sequences 1 and 2 (VDCS-1or 2) are indicated.
