# Supplementary material for: Genome Sequence of Babesia bovis and Comparative Analysis of Apicomplexan Hemoprotozoa
Source: PLoS Pathog. 2007 Oct 19;3(10):e148. doi: 10.1371/journal.ppat.0030148 (PMC2034396; doi:10.1371/journal.ppat.0030148)
Supplement: Supplementary file 4 [file ppat.0030148.sg004.pdf]

Supplementary Figure 4:

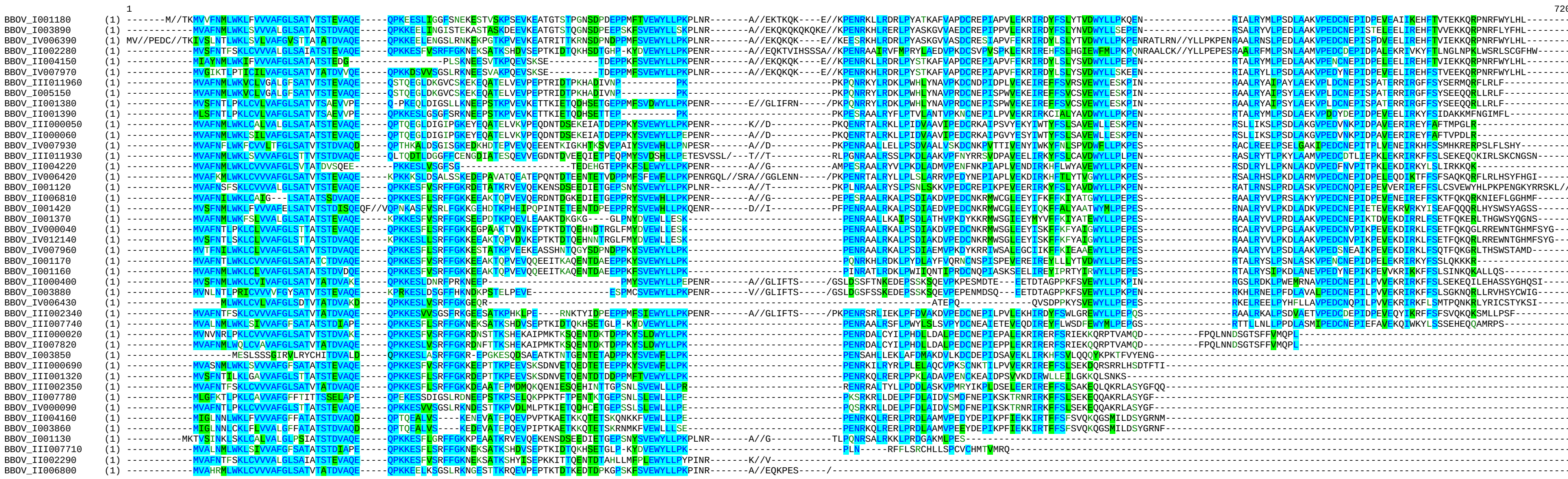

Supplementary Figure 4: Alignment of 44 SmORF sequences. Deduced amino acid sequences were aligned with the AlignX module of VectoNTI. The blue background indicates positions with identical amino acid sequences, while the green background indicates conserved amino acids. Dashes indicate that there is no amino acid in that position. Long stretches of intervening amino acid sequence has been trimmed from a few sequences to allow visualization of the four blocks of amino acid conservation. The double slashes (//) indicate that sequence was trimmed from this position that spanned the full length between the remaining amino acids, while a single slash (/) indicates that sequence the sequence that was trimmed from the alignment that did not fully span the region that was removed. The total alignment length is 720 amino acids in length.
