# Supplementary material for: Genome Sequence of Babesia bovis and Comparative Analysis of Apicomplexan Hemoprotozoa
Source: PLoS Pathog. 2007 Oct 19;3(10):e148. doi: 10.1371/journal.ppat.0030148 (PMC2034396; doi:10.1371/journal.ppat.0030148)
Supplement: Supplementary file 5 [file ppat.0030148.sg005.pdf]

Supplementary Figure 5:

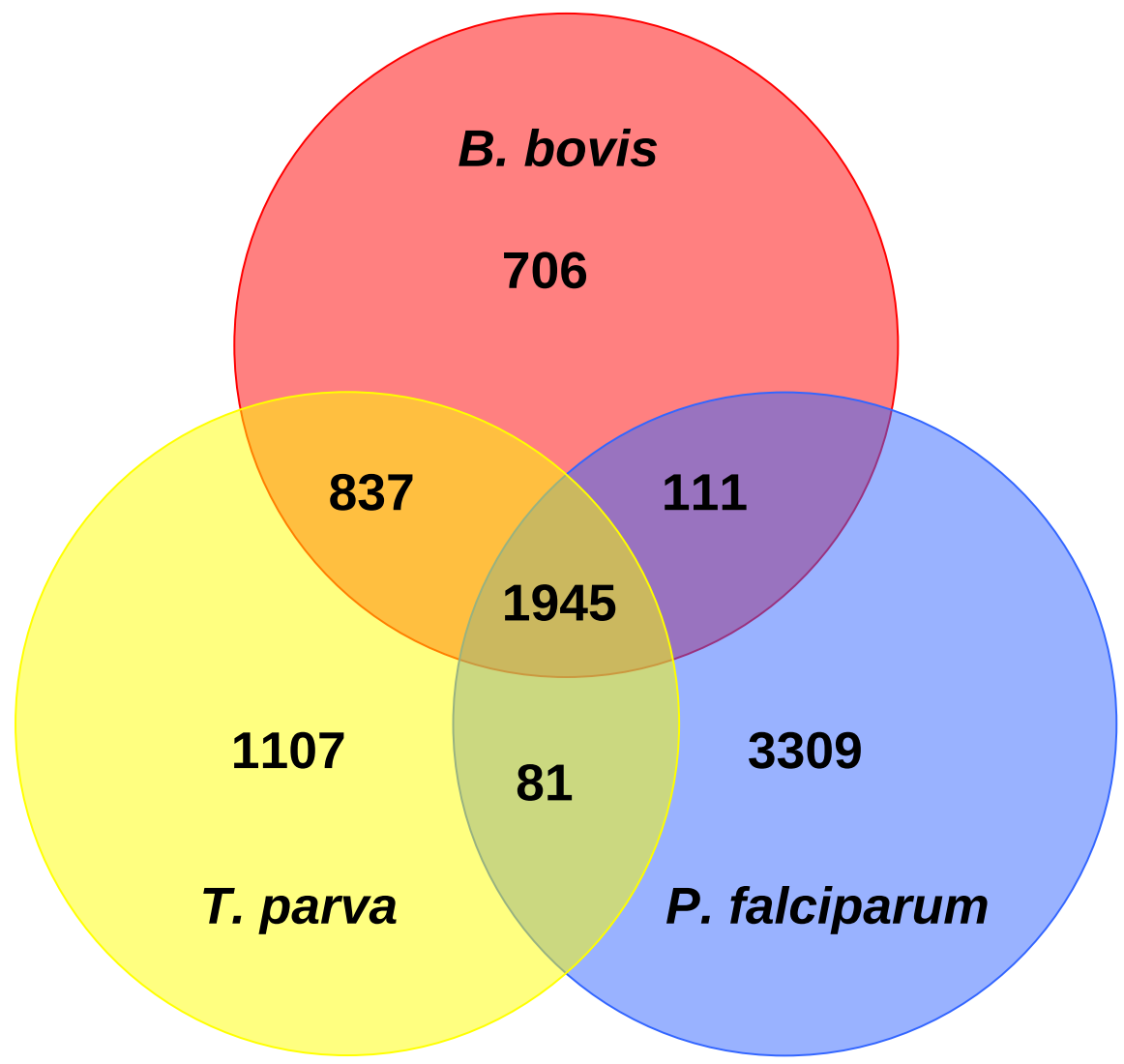

Supplementary Figure 5: Venn diagram showing number of genes in overlapping COGs between *B. bovis*, *T. parva* and *P. falciparum*.
