# Supplementary material for: Genome Sequence of Babesia bovis and Comparative Analysis of Apicomplexan Hemoprotozoa
Source: PLoS Pathog. 2007 Oct 19;3(10):e148. doi: 10.1371/journal.ppat.0030148 (PMC2034396; doi:10.1371/journal.ppat.0030148)
Supplement: Supplementary file 6 [file ppat.0030148.sg006.pdf]

Supplementary Figure 6:

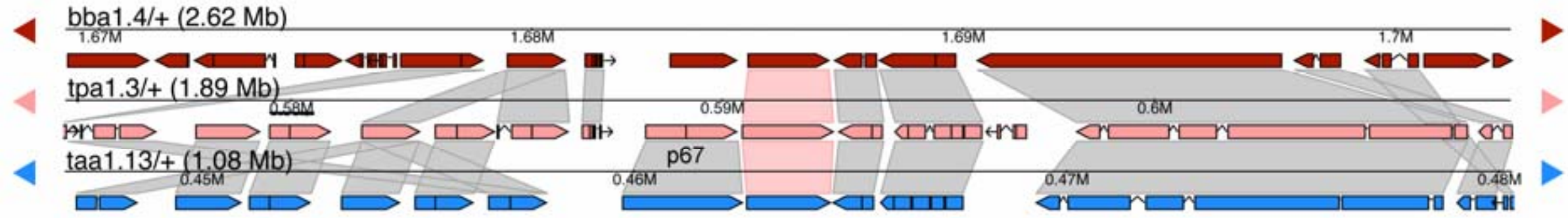

Supplementary Figure 6: Example of microsynteny between *B. bovis* and *T. parva*. The *T. parva* p67 locus is diagrammed in the middle row, with the corresponding *B. bovis* locus shown on the top row, and the *T. annulata* SPAG1 locus diagrammed on the bottom row. Genes with sequence identity between species are connected by shaded gray lines. The gene highlighted with pink was used to identify the syntenic locus. The p67 gene is indicated
