# Supplementary material for: Genome Sequence of Babesia bovis and Comparative Analysis of Apicomplexan Hemoprotozoa
Source: PLoS Pathog. 2007 Oct 19;3(10):e148. doi: 10.1371/journal.ppat.0030148 (PMC2034396; doi:10.1371/journal.ppat.0030148)
Supplement: Supplementary file 7 [file ppat.0030148.st001.doc]

Supplementary Table 1: Transporters

|  | ***B. bovis T2Bo*** | ***C. parvum genotype 2 isolate*** | ***P. falciparum 3D7*** | ***T. parva*** |
| --- | --- | --- | --- | --- |
| **Genome Size(Mb):** | 8.2 | 9.1 | 23 | 8.4 |
| **Total Transporter Proteins:** | 66 | 75 | 87 | 74 |
| **No. of Transporters per Mb genome:** | **8.25** | **8.24** | **3.78** | **8.81** |
|  | | | | |
| **ATP-Dependent** | **20 (30%)** | **23 (31%)** | **30 (34%)** | **28 (38%)** |
| The ATP-binding Cassette (ABC) Superfamily | 9 | 13 | 14 | 17 |
| The Arsenite-Antimonite (ArsAB) Efflux Family | 1 | 0 | 1 | 1 |
| The H+- or Na+-translocating F-type, V-type and A-type ATPase (F-ATPase) Superfamily | 2 | 1 | 2 | 2 |
| The H+-translocating Pyrophosphatase (H+-PPase) Family | 0 | 0 | 2 | 1 |
| The P-type ATPase (P-ATPase) Superfamily | 8 | 9 | 11 | 7 |
|  | | | | |
| **Ion Channels** | **6 (9%)** | **5 (7%)** | **6 (7%)** | **4 (5%)** |
| The Chloride Channel (ClC) Family | 0 | 0 | 0 | 0 |
| The Epithelial Na+ Channel (ENaC) Family | 0 | 0 | 0 | 0 |
| The Major Intrinsic Protein (MIP) Family | 0 | 0 | 1 | 0 |
| The CorA Metal Ion Transporter (MIT) Family | 0 | 0 | 2 | 0 |
| The Small Conductance Mechanosensitive Ion Channel (MscS) Family | 2 | 2 | 1 | 2 |
| The Non-selective Cation Channel-2 (NSCC2) Family | 1 | 1 | 1 | 1 |
| The Voltage-gated Ion Channel (VIC) Superfamily | 3 | 2 | 1 | 1 |
| **Secondary Transporter** | **39 (59%)** | **42 (56%)** | **49 (56%)** | **41 (55%)** |
| The Amino Acid/Auxin Permease (AAAP) Family | 0 | 7 | 2 | 0 |
| The Ca2+:Cation Antiporter (CaCA) Family | 0 | 2 | 1 | 0 |
| The Cation Diffusion Facilitator (CDF) Family | 1 | 1 | 1 | 0 |
| The Monovalent Cation:Proton Antiporter-1 (CPA1) Family | 2 | 0 | 1 | 1 |
| The Monovalent Cation:Proton Antiporter-2 (CPA2) Family | 0 | 1 | 0 | 0 |
| The Dicarboxylate/Amino Acid:Cation (Na+ or H+) Symporter (DAACS) Family | 0 | 0 | 0 | 1 |
| The Divalent Anion:Na+ Symporter (DASS) Family | 0 | 0 | 0 | 0 |
| The Drug/Metabolite Transporter (DMT) Superfamily | 7 | 11 | 6 | 4 |
| The Equilibrative Nucleoside Transporter (ENT) Family | 0 | 1 | 2 | 0 |
| The Folate-Biopterin Transporter (FBT) Family | 1 | 1 | 2 | 3 |
| The Formate-Nitrite Transporter (FNT) Family | 1 | 0 | 1 | 1 |
| The Glycoside-Pentoside-Hexuronide (GPH):Cation Symporter Family | 0 | 0 | 1 | 0 |
| The Glycerol Uptake (GUP) Family | 0 | 0 | 0 | 0 |
| The Lysosomal Cystine Transporter (LCT) Family | 0 | 1 | 0 | 0 |
| The Mitochondrial Carrier (MC) Family | 7 | 5 | 10 | 9 |
| The Major Facilitator Superfamily (MFS) | 14 | 8 | 15 | 16 |
| The Mitochondrial Tricarboxylate Carrier (MTC) Family | 0 | 0 | 0 | 0 |
| The Metal Ion (Mn2+-iron) Transporter (Nramp) Family | 0 | 0 | 1 | 0 |
| The Neurotransmitter:Sodium Symporter (NSS) Family | 1 | 0 | 1 | 1 |
| The Cytochrome Oxidase Biogenesis (Oxa1) Family | 1 | 0 | 1 | 1 |
| The Inorganic Phosphate Transporter (PiT) Family | 1 | 0 | 1 | 1 |
| The Resistance-Nodulation-Cell Division (RND) Superfamily | 0 | 2 | 1 | 0 |
| The Sulfate Permease (SulP) Family | 1 | 0 | 1 | 1 |
| The Zinc (Zn2+)-Iron (Fe2+) Permease (ZIP) Family | 2 | 2 | 1 | 2 |
|  | | | | |
| **Unclassified** | **1 (2%)** | **5 (7%)** | **2 (2%)** | **1 (1%)** |
| The Copper Transporter-1 (Ctr1) Family | 0 | 0 | 0 | 0 |
| The Copper Transporter-2 (Ctr2) Family | 1 | 1 | 2 | 1 |
| The Peptide Uptake Permease (PUP) Family | 0 | 4 | 0 | 0 |
