# Supplementary material for: Genome Sequence of Babesia bovis and Comparative Analysis of Apicomplexan Hemoprotozoa
Source: PLoS Pathog. 2007 Oct 19;3(10):e148. doi: 10.1371/journal.ppat.0030148 (PMC2034396; doi:10.1371/journal.ppat.0030148)
Supplement: Supplementary file 8 [file ppat.0030148.st002.doc]

Supplementary Table 2: Characteristics of apicomplexan plastids

|  | Pf^1^ | Tg^2^ | Et^3^ | Tp^4^ | Bb^5^ |
| --- | --- | --- | --- | --- | --- |
| Genome Size (bp) | 34,682 | 34,996 | 34,750 | 39,579 | 33,351 |
| # of putative protein-coding genes | 30 | 28 | 28 | 44 | 32 |
| # of tRNA genes | 34 | 33 | 32 | 30 | 25 |
| # of rRNA genes | 4 | 4 | 4 | 2 | 2 |
| A+T(%) | 86.9 | 78.5 | 79.4 | 80.5 | 78.2 |
| Coding strands | BS^6^ | BS | BS | SS^7^ | SS |

^1^Pf=*Plasmodium falciparum*; ^2^Tg=*Toxoplasma gondii*; ^3^Et=*Eimeria tenella*;

^4^Tp=*Theileria parva*; ^5^Bb=*Babesia bovis*; ^6^BS=both strands; ^7^SS=single strand.
