# Supplementary material for: Genome Sequence of Babesia bovis and Comparative Analysis of Apicomplexan Hemoprotozoa
Source: PLoS Pathog. 2007 Oct 19;3(10):e148. doi: 10.1371/journal.ppat.0030148 (PMC2034396; doi:10.1371/journal.ppat.0030148)
Supplement: Supplementary file 9 [file ppat.0030148.st003.doc]

Supplementary Table 3: Nuclear encoded genes potentially targeted to the apicoplast

| Gene identifier | Functional name | SP^a^ | Pathway^b^ | AP^c^ | Extension^d^ | TP^e^ |
| --- | --- | --- | --- | --- | --- | --- |
| BBOV_I000120 | farnesyl pyrophosphate synthetase, putative | - | + | - | - | - |
| BBOV_III000400 | 4-hydroxybenzoate octaprenyltransferase | - | + | - | + | - |
| BBOV_III001660 | LytB protein | + | + | - | + | + |
| BBOV_III002600 | 1-deoxy-D-xylulose 5-phosphate synthase family protein | - | + | - | + | + |
| BBOV_III003490 | 2-C-methyl-D-erythritol 4-phosphate cytidylyltransferase | - | + | - | + | + |
| BBOV_III010740 | 1-deoxy-D-xylulose 5-phosphate reductoisomerase family protein | - | + | - | + | + |
| BBOV_II006930 | 4-hydroxy-3-methylbut-2-en-1-yl diphosphate synthase (gcpE protein) | + | + | - | + | + |
| BBOV_IV002810 | 2-C-methyl-D-erythritol 2,4-cyclodiphosphate synthase, putative | - | + | - | + | + |
| BBOV_I000160 | conserved hypothetical protein | + | - | + | NH^f^ | + |
| BBOV_I000990 | hypothetical protein | + | - | + | NH | - |
| BBOV_III005720 | tRNA pseudouridine synthase, putative | + | - | + | + | + |
| BBOV_III010050 | ubiquitin family protein | + | - | + | - | - |
| BBOV_III011070 | hypothetical protein | + | - | + | NH | - |
| BBOV_II006740 | ubiquitin-conjugating enzyme E2 | + | - | + | + | - |
| BBOV_II005780 | membrane protein, putative | + | - | + | NH | + |
| BBOV_I001660 | hypothetical protein | + | - | + | NH | - |
| BBOV_IV001370 | MAC/perforin domain containing protein | + | - | + | NH | + |
| BBOV_IV005750 | membrane protein, putative | + | - | + | NH | + |
| BBOV_IV010950 | tyrosyl-tRNA synthetase, putative | + | - | + | + | + |
| BBOV_III000740 | hypothetical protein | +/- | - | + | - | + |
| BBOV_III008780 | membrane protein, putative | + | - | + | NH | + |
| BBOV_IV000480 | 50S ribosomal protein L33 | +/- | - | + | + | + |
| BBOV_I000970 | DnaJ domain containing protein | + | - | - | + | - |
| BBOV_I000700 | ribosomal protein S14p/S29e domain containing protein | - | - | - | + | + |
| BBOV_I000150 | conserved hypothetical protein | - | - | - | + | - |
| BBOV_I000200 | conserved hypothetical protein | + | - | - | + | - |
| BBOV_I001000 | PET112 family, N terminal region domain containing protein | + | - | - | + | + |
| BBOV_I001010 | tRNA modification GTPase TrmE , putative | +/- | - | - | + | + |
| BBOV_III000580 | malate:quinone oxidoreductase, putative | +/- | - | - | + | - |
| BBOV_III005900 | apurinic endonuclease (APN1) family protein | - | - | - | + | + |
| BBOV_III006230 | uracil-DNA glycosylase family protein | - | - | - | + | - |
| BBOV_III001040 | conserved hypothetical protein | - | - | - | + | + |
| BBOV_III007790 | casein kinase II, alpha chain (CK II) | + | - | - | + | - |
| BBOV_III008320 | 50S ribosomal protein L3, putative | - | - | - | + | - |
| BBOV_III009120 | protein kinase domain containing protein | - | - | - | + | - |
| BBOV_III002510 | conserved hypothetical protein | - | - | - | + | NH |
| BBOV_III002530 | tryptophanyl-tRNA synthetase family protein | - | - | - | + | - |
| BBOV_IV000250 | glucosamine--fructose-6-phosphate aminotransferase | - | - | - | + | - |
| BBOV_IV000910 | S-adenosyl methyltransferase, putative | + | - | - | + | + |
| BBOV_IV000300 | Der1-like family, putative | - | - | - | + | NH |
| BBOV_IV003600 | ribosomal protein L7/L12 | - | - | - | + | - |
| BBOV_IV001210 | conserved hypothetical protein | - | - | - | + | - |
| BBOV_IV005700 | 50S ribosomal protein L13 ,putative | - | - | - | + | - |
| BBOV_IV003870 | molybdenum cofactor synthesis protein 3 / molybdopterin synthase sulphurylase, putative | - | - | - | + | - |
| BBOV_IV006630 | methionyl-tRNA synthetase, putative | - | - | - | + | + |
| BBOV_IV004710 | translation elongation factor G, putative | + | - | - | + | + |
| BBOV_IV010190 | ribosomal protein L1, putative | - | - | - | + | - |

^a^ Has a signal peptide as predicted by SignalP. +/- indicates was positive by only 1 algorithm.

^b^ Belongs to a pathway predicted to be in the apicoplast

^c^ Contains an apicoplast targeting domain as predicted by PlasmoAP

^d^ Has an amino-terminal extension when compared to orthologs from non-apicoplast containing organisms. NH indicates no orthologs amongst non-apicoplast containing organisms.

^e^ *Theileria parva* ortholog is listed as potentially targeted to the apicoplast. NH indicates no ortholog in *T. parva*.
